# Highly Efficient Synthesis of an Emerging Lipophilic Antioxidant: 2-Ethylhexyl Ferulate

**DOI:** 10.3390/molecules21040478

**Published:** 2016-04-12

**Authors:** Kuo-Chuan Huang, Ying Li, Chia-Hung Kuo, Yawo-Kuo Twu, Chwen-Jen Shieh

**Affiliations:** 1 Department of BioIndustry Technology, Da-Yeh University, 168 University Road, Dacun, Changhua 51591, Taiwan; freedomlove0216@gmail.com (K.-C.H.); poly2001@mail.dyu.edu.tw (Y.-K.T.); 2 Biotechnology Center, National Chung Hsing University, 250 Kuokuang Road, Taichung 40227, Taiwan; quatrefoil47@yahoo.com.tw; 3 Department of Seafood Science, National Kaohsiung Marine University, 142 Haijhuan Road, Nanzih District, Kaohsiung 81157, Taiwan; chkuo@webmail.nkmu.edu.tw

**Keywords:** 2-ethylhexyl ferulate, lipase, reduced pressure evaporation system, response surface methodology, artificial neural network

## Abstract

Ferulic acid in ester form has shown a stronger ability in ameliorating certain pathological conditions and inhibiting lipid oxidation. In present study, a solvent-free and reduced pressure evaporation system was developed for lipase-catalyzed synthesis of 2-ethylhexyl ferulate (2-EF) from ferulic acid and 2-ethylhexanol. A Box-Behnken design with response surface methodology (RSM) and artificial neural network (ANN) was selected to model and optimize the process. Based on the yields of 2-EF, reaction temperature was shown to be the most important process factor on the molar conversion among all variables. The residual values and the coefficient of determination (*R*^2^) calculated from the design data indicated that ANN was better than RSM in data fitting. Overall, the present lipase-catalyzed approach for 2-EF synthesis at low reaction temperature in a reduced pressure evaporation system shows high 2-EF production efficiency. Notably, this approach can reduce the enzyme denaturation and ferulic acid oxidation that usually occur during long-term biosynthetic operations at high temperature.

## 1. Introduction

Ferulic acid (FA) belongs to the family of phenolic acids and is very abundant in grains, fruits and vegetables. Studies have shown that FA displays excellent antioxidant [1], ultraviolet-absorbing activities [2,3] and potential health benefits against cardiovascular problems, inflammatory diseases and cancer [4]. However, the solubility of FA in hydrophobic solvents is low, thus limiting its utilization in the pharmaceutical, cosmetic and food industries. In order to increase utilization of FA, lipophilization is needed, where enzymatic esterification with alcohols has proved to be a promising approach [5,6,7,8]. In recent years, many studies have report that the enzymatic synthesis of hydrophobic derivatives of FA can increase its oil-solubility [9,10,11], and some of them have been applied for preparation of emulsion-based creams due to their anti-inflammatory activity [12]. FA esters, the hydrophobic derivatives of FA, have also demonstrated other bioactivities such as antioxidant [13], anticonvulsant [14], synergistic photobactericidal effects [15], and antithrombotic activities [16], as well as inhibition of the atherogenic effects of leptin [17]. Interestingly, FA esters possess better inhibitory effect on lipid oxidation than FA [18].

Enzymatic biosynthesis of these compounds has attracted much attention in recent years, and it may be a better approach in comparison to conventional chemical processes [19,20,21,22,23]. It has been reported that continuous synthesis of alkyl ferulate by immobilized lipase shows great conversion rates (approximately 90%) at 90 °C after two days [21]. It should be kept in mind that FA is susceptible to oxidation under high temperatures. Novozym^®^435 had been used in the lipase-catalyzed synthesis of ferulate esters, but the yield was only 17% after several days [22]. Generally, solvent-free reaction systems are applied in the lipase-catalyzed synthesis of ferulate esters to decrease the reaction time and increase the yield [20,23]. The solvent-free system is a simple mixture of substrates, offering advantages including maximization of substrate concentration, greater volumetric production, and cost savings in reactor design and product separation. In addition, the boiling point of the component liquids is lower in a reduced pressure evaporation system than that in the atmosphere at the same temperature, suggesting that the byproducts with relative low vapor pressure physical characteristics are more prone to be eliminated in the reduced pressure system. This may increase the efficiency of the lipase-catalyzed reaction. Therefore, a reduced pressure evaporation system combined with a solvent-free system may be an excellent strategy to overcome the problems of long reaction time and low yields of FA esters synthesis. So far, the literature regarding the lipase-catalyzed esterification of ferulic acid in a reduced pressure evaporation system is still limited. Notably, there have been no reports regarding lipase-catalyzed synthesis of 2-ethylhexylferulate (2-EF), a common FA ester with great inhibitory effect on lipid oxidation, which has been widely used as the antioxidant in the food and cosmetic industries.

Recently, response surface methodology (RSM) and an artificial neural network (ANN) approach have been applied for optimization and process modeling. The development of an optimum enzymatic synthesis procedure to improve the yield conversion to reduce production costs would be attractive for manufacturers and consumers. RSM and ANN have been successfully applied to model and optimize extraction of lignans [24], curdlan production [25], and biodiesel production [26]. In this study, a reduced pressure evaporation system was used to synthesize 2-ethylhexyl ferulate (2-EF) under solvent free conditions. RSM and ANN were employed to investigate the effects of different reaction variables (reaction time, reaction temperature, and enzyme amount) on the response (yield %), and to obtain the optimal conditions to solve the problems of long reaction time and low yield of FA esters.

## 2. Results and Discussion

The present study applied RSM and ANN for experimental design of enzymatic synthesis of 2-EF in a solvent-free system. The molar ratio of FA and 2-ethylhexanol (the reactants) was 1:129, and the independent parameters selected for the experimental design were: reaction time (X_1_), enzyme amount (X_2_), and reaction temperature (X_3_). Their ranges and levels are given in Table 1.

### 2.1. RSM Model

The RSM Box-Behnken procedure was employed to fit the second-order polynomial equation to the experimental data (Table 1). Among the various treatments, the highest molar conversion (98.9% ± 2.5%) was observed in treatment 12 (reaction temperature of 80 °C, enzyme amount of 1500 PLU and reaction time of 16 h), and the lowest molar conversion (42.7% ± 3.3%) was observed in treatment 10 (reaction temperature of 60 °C, enzyme amount of 1000 PLU and reaction time of 8 h). The second-order polynomial equation obtained was as follows:
Y = −968.26667 + 3.60052X_1_ + 26.28208X_2_ + 0.039783X_3_ − 0.027812X_1_X_2_ + 0.0003X_1_X_3_ +0.0004X_2_X_3_ − 0.028971X_1_^2^ − 0.17154X_2_^2^ − 0.0000305167X_3_^2^


The analysis of variance (ANOVA), represented in Table 2, indicates that this second-order polynomial model was highly significant and adequate to represent the actual relationship between the response (molar conversion) and the significant variables with a very small *p* value (<0.001) and a satisfactory coefficient of determination (*R*^2^ = 0.9852). The ANOVA results of responses reveal an insignificant “lack of fit” for *p* > 0.05. Therefore, these models were adequate for prediction within the range of variables employed. Furthermore, the ANOVA results indicate that the linear term of X_1_ and X_3_ and the square term of X_2_^2^ and X_3_^2^ had significant (*p* < 0.05) influence on molar conversion. The interaction terms had less influence (*p* > 0.05).

**Figure 1:**
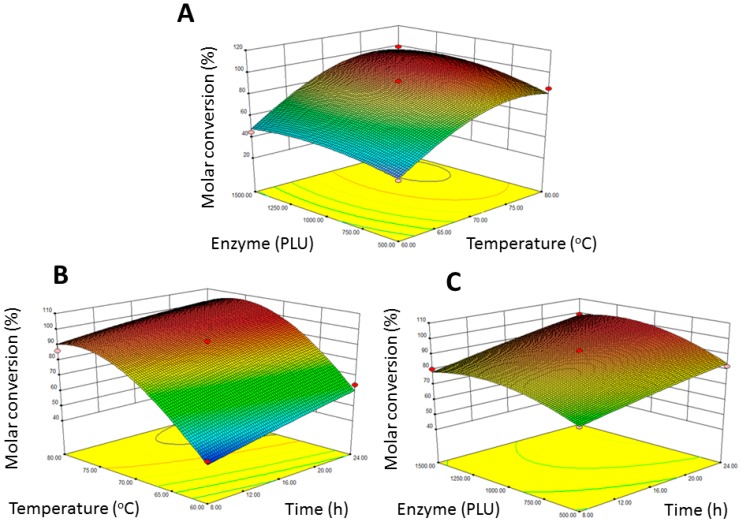
Response surface plot for the relationships between molar conversion and experimental variables. Response surface plot showing the relationships between molar conversion of 2-ethylhexyl ferulate and reaction parameters: (**A**) reaction temperature and enzyme amount; (**B**) reaction temperature and reaction time and (**C**) reaction time and enzyme amount.

The effect of varying reaction temperature and enzyme amount on transesterification efficiency at a constant reaction time of 16 h was shown in Figure 1A. With the lowest reaction temperature (60 °C) and enzyme amount (500 PLU), a 2-EF molar conversion of 39.8% was obtained. Low molar conversions are also found in a previous report on lipase-catalyzed synthesis of ferulate esters at the temperature of 60 °C, enzyme amount of 100 mg, and time of 240 h [22]. The low molar conversion of ferulate esters can be improved by using a reduced pressure evaporation system. In the reduced pressure evaporation system, when the enzyme amount was increased to 1500 PLU and the reaction temperature was increased to 80 °C, the molar conversion of 2-EF was increased to 98.9 (Figure 1A). The great increase in molar conversion can result from the impact of solvent-free and reduced pressure evaporation system which increases the reaction rate of lipase-catalyzed biosynthesis by elevating the substrate concentration and eliminating byproducts with low vapor pressure physical characteristics. Figure 1B shows the effects of reaction time and reaction temperature on 2-EF biosynthesis at a constant enzyme amount (1000 PLU). At the lowest reaction time (8 h) and reaction temperature (60 °C), the molar conversion was 42.7% ± 3.3%. Figure 1C shows the effects of reaction time and enzyme amount on 2-EF biosynthesis at a constant reaction temperature. In this regard, enzyme amount had an insignificant effect on molar conversion, while reaction time had only a partial effect on molar conversion. Taken together, the molar conversion significantly increased when the reaction temperature and reaction time increased. As the ANOVA results shown in Table 2 indicate, reaction temperature and reaction time are important factors in Novozym^®^435-catalyzed reactions, which is in accordance with our previous works [27]. However, it has been reported that higher reaction temperature might decrease the lipase activity [21]. Thus, a reduced pressure evaporation system can be a good solution for lipase-catalyzed synthesis of FA esters in order to obtain a higher conversion at a lower reaction temperature.

### 2.2. ANN Model

ANN is a promising modeling methodology for non-linear multivariate process/reaction [28]. In this study, the data generated from the Box-Behnken design (Table 1) were employed to generate an ANN model for the enzymatic 2-EF synthesis. The proposed ANN consisted of three layers in the present work: an input layer with four neurons (reaction time, reaction temperature, and enzyme amount), a hidden layer with several neurons and an output layer containing one output neuron (molar conversion). The feed-forward neural network was trained by using incremental back propagation (IBP), batch backpropagation (BBP) and Levenberg-Marquardt algorithm (LM) or genetic algorithm (GA), respectively. All neurons from the hidden layer and output layer were calculated by a sigmoid transfer function. Different numbers of neurons, from three to six, were examined. The various architectures for ANN models were developed using NeuralPower software (Version 2.5, CPC-X Software, USA; available from Internet: http://www.brothersoft.com/neuralpower-download-21356.html), and the best one was selected based on the maximization of the *R*^2^ value and the minimization of the RMSE value.

**Figure 2:**
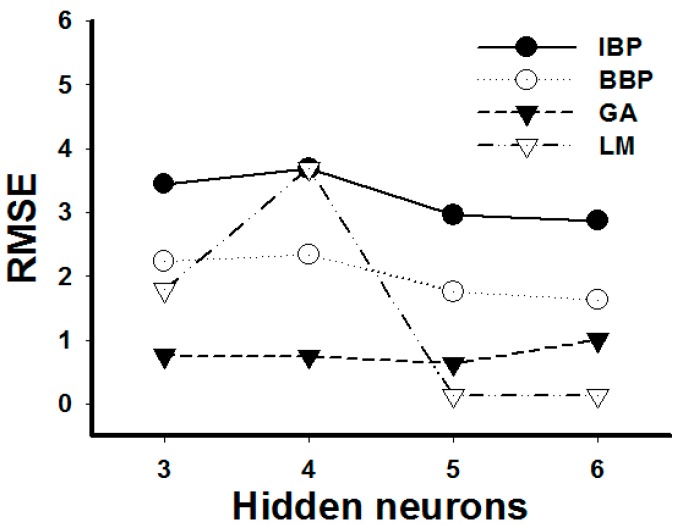
Learning curves of ANN model at different hidden neurons. The training set uses sigmoid transfer function in the hidden layer and output layer based on increment back propagation (IBP), batch back propagation (BBP), Levenberg-Marquardt algorithm (LM) or genetic algorithm (GA).

**Figure 3:**
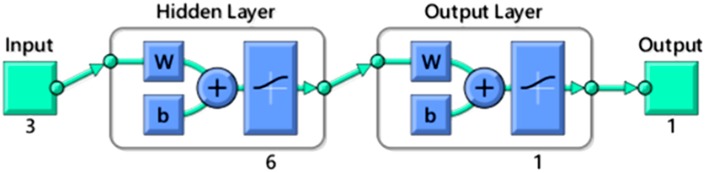
The optimal architecture of multilayer feed forward neural network. The network contains three inputs (reaction temperature, reaction time and enzyme amount), one hidden layer with six nodes and one output (molar conversion of 2-ethylhexyl ferulate). W means weight; b means basic input/output system (BIOS).

All results from the test were evaluated statistically based on the RMSE values. According to Figure 2, the best performance network was trained using a LM algorithm with a sigmoid transfer function, which exhibited a lower and stable RMSE. It is noted that the curve of the RMSE levels off at five hidden neurons. Therefore, the best ANN model in this study is determined to be a multilayer feed forward connection, trained by a LM algorithm using a sigmoid transfer function that consists of a 3–6–1 topology, as shown in Figure 3. This learning was acquired with RMSE = 0.79, *R*^2^ = 0.99.

**Figure 4:**
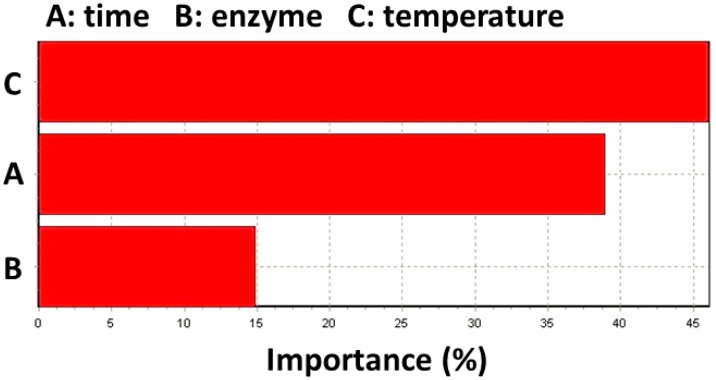
Importance of experimental variables on lipase-catalyzed synthesis of 2-ethylhexyl ferulate.

According to Figure 4, the reaction time and reaction temperature displayed great impact on the conversion with a relative importance of approximate 39% and 46%, respectively. These results are in accordance with the ones obtained with RSM.

### 2.3. Comparison between RSM and ANN and Optimization for Experimental Condition

**Figure 5:**
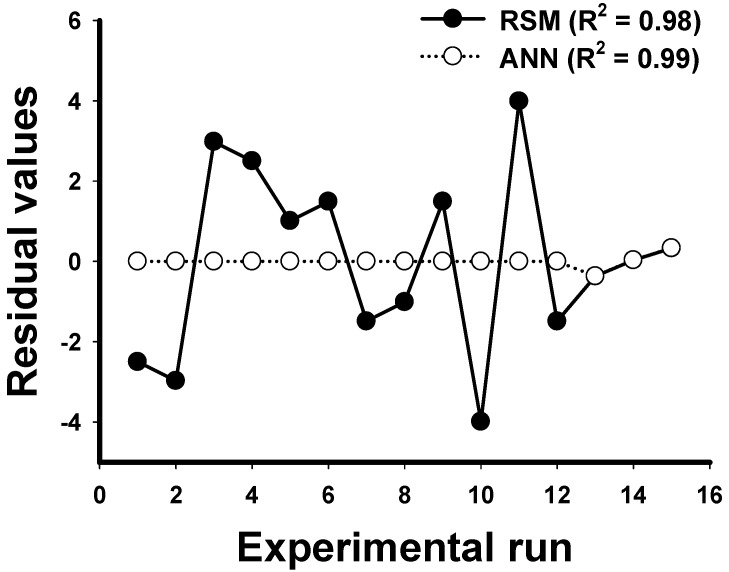
The distribution of residual values for each experimental run of Box-Behnken design experiments employing the RSM and ANN models. Coefficient of determination (*R*^2^) calculated from the design data for RSM and ANN models were 0.98 and 0.99, respectively. Residual values: predicted values minus actual values.

The distribution of the residual values obtained from the experimental data of Box-Behnken design experiments minus the related computed RSM and ANN data are shown in Figure 5. It was observed that the deviations of residual values showed a relatively small variation in the ANN model when compared with the RSM model. This suggested that ANN was better than RSM in data fitting.

In order to validate and test the extrapolative capability of both the ANN and RSM models, a completely new set of five experiments was conducted on an experimental range which does not belong to the design data sets. The experimental and predicted values of the response for both the ANN and RSM models are given in Table 3. The performance of the newly constructed ANN and RSM models were statistically measured, in terms of AAD and RMSE values. The RMSEs for RSM and ANN were found as 7.322 and 5.152. These results indicate that the RSM prediction has a greater deviation than the ANN prediction. The AAD for RSM and ANN was found to be 10.046 and 9.430. From the results, it was observed that predictive power of ANN was found to be more powerful than RSM.

Ridge max analysis can be used to determine optimum operating conditions by computing the estimated ridge of the maximum response for increasing the radius from the center of the original design. As shown in Table 4, the optimum condition for lipase-catalyzed synthesis of 2-EF determined by the ridge max analysis of RSM was reaction time of 23 h, reaction temperature of 71 °C and enzyme amount of 1422 PLU. A verification experiment performed at this condition obtained a molar conversion of 99.43%. Notably, higher molar conversion (99.74) was observed when the experiment was performed using the ANN-modeled optimal conditions (Table 4).

### 2.4. Evaluation of Enzyme Reusability

**Figure 6:**
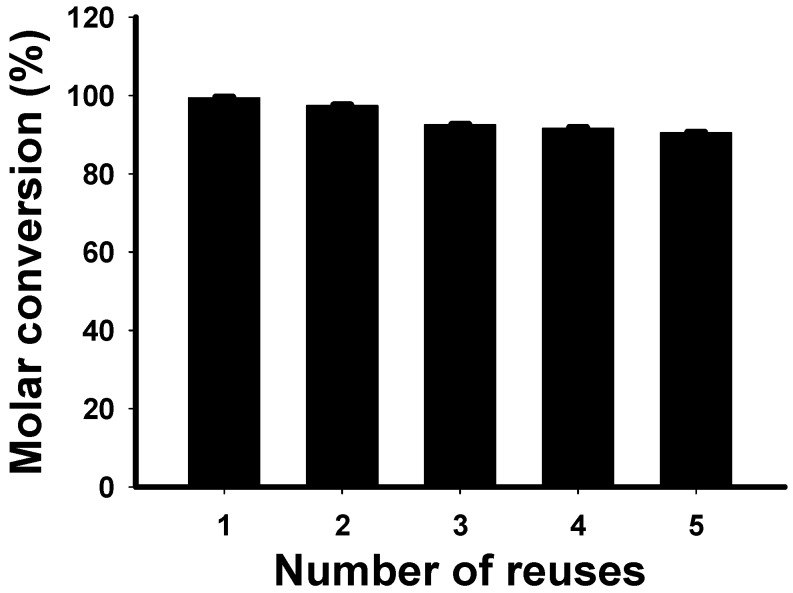
Reusability of Novozym^®^435 in the synthesis of 2-ethylhexyl ferulate performed under optimum conditions.

To examine the enzyme reusability, the ability of reused immobilized lipase for 2-EF synthesis was investigated under optimum conditions (Figure 6). After reaction, the mixture was filtered through a 0.45 μm membrane. The immobilized lipase was then recovered from the membrane, washed three times with fresh reaction media and dried before reuse in the next batch. After the immobilized lipase was reused five times, the molar conversion of 2-EF still remained at about 90.4%. This result showed that the immobilized lipase remained stable through long-term 2-EF exposure. Thus, the result demonstrated that the lipase could be effectively applied for 2-EF synthesis in the reduced pressure evaporation system and that the stability was high enough to permit reuse.

## 3. Experimental Section

### 3.1. Materials

Immobilized lipase Novozym^®^435 (10,000 U/g; propyl laurate units (PLU)) from *Candida antarctica* B (EC3.1.1.3) supported on a macroporous acrylic resin was purchased from NovoNor disk Bioindustrials Inc. (Copenhagen, Denmark). Ferulic acid (FA), 2-ethylhexanol, 2-methyl-2-butanol, methanol and acetic acid were purchased from the Sigma Chemical Co. (St. Louis, MO, USA). A 4 Å molecular sieve was purchased from Davison Chemical (Baltimore, MD, USA). 2-ethylhexyl ferulate (2-EF) was purchased from Shanghai Richem International Co., Ltd. (Shanghai, China). All the other reagents and chemicals, unless otherwise noted, were of analytical grade.

### 3.2. Enzymatic 2-ethylhexyl Ferulate Synthesis

**Scheme 1:**
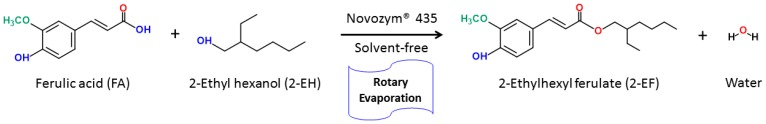
Enzymatic synthesis of 2-ethylhexyl ferulate.

FA was dissolved in 2-ethylhexanol (1 mL) to the concentration of 0.05 M in the fingernails emissions glass flask that contained Novozym^®^435 of a rotary evaporator (EYELA N-1100, Tokyo Rikakikai Co., Ltd., Bohemia, NY, USA), which operated at 80 rpm and 560 torr under different experimental conditions. The esterification of FA with 2-EF catalyzed by Novozym^®^435 is represented in Scheme 1.

**Figure 7:**
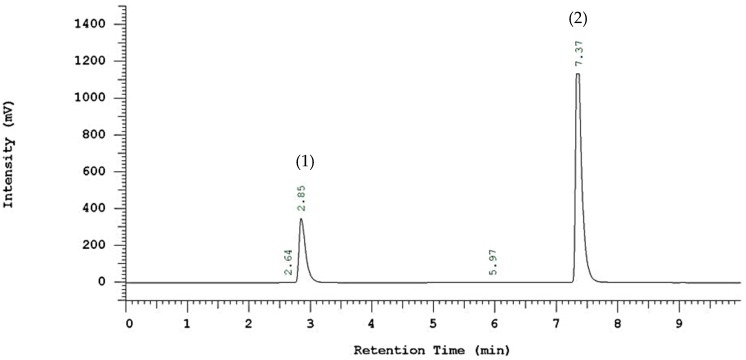
HPLC chromatogram of ferulic acid and its ester form. (1) ferulic acid; (2) 2-ethylhexyl ferulate.

After incubation, liquid samples were withdrawn from the reaction mixture for analysis of 2-EF by high-performance liquid chromatography (HPLC). The sample was diluted with hexanol/2-methyl-2-butanol (1:150), and was injected (30 μL) into a HPLC (Hitachi L-7400; Tokyo, Japan) equipped with UV detector and Thermo C18 column (250 mm × 4.6 mm, Agilent, Santa Clara, CA, USA). Separations were carried out by a gradient elution with 0.1% acetic acid and methanol, the flow rate was set to 1.0 mL/min, and 2-EF was detected under UV light at 325 nm as shown in Figure 7. Calibration curves were prepared from the FA and 2-EF standards based on the peak areas. The molar conversion was defined as (mmol of 2-EF production per mmol of initial FA) × 100%.

### 3.3. Box-Behnken Design

A Box-Behnken design for 15 experimental runs was employed in present study. To avoid bias, the 15 runs were performed in a totally random order. The variables and their levels selected for the study of 2-EF biosynthesis were: reaction temperature (60 °C–80 °C), enzyme amount (500–1500 PLU) and reaction time (8–24 h), which were coded as shown in Table 5. Table 1 shows the independent factors (xi), levels and experimental design. Each experimental point was carried out in duplicate. Statistical parameters mentioned in present study including coefficient of determination (*R*^2^), root-mean-square error (RMSE), and absolute average deviation (AAD) were calculated by following Equations (1) to (3):

(1) $$R^{2}=1-\frac{\sum_{i=1}^{n}{\left(Y_{pre}-Y_{exp}\right)}^{2}}{\sum_{i=1}^{n}{\left(Y_{m}-Y_{exp}\right)}^{2}}$$

where *Y_pre_* is the predicted 2-EF yield (by either RSM or ANN), *Y_exp_* is the observed 2-EF yield and *Y_m_* is the average 2-EF yield.

(2) $$RMSE=\sqrt{\frac{\sum_{i=1}^{n}{\left(Y_{pre}-Y_{exp}\right)}^{2}}{n}}$$

where *Y_pre_* and *Y_exp_* are the predicted and experimental response, respectively, and *n* is the number of experiments.

(3) $$AAD=\left[\frac{\sum_{i=1}^{n}\left(\left|Y_{exp}-Y_{pre}\right|/Y_{pre}\right)}{n}\right]\times 100$$

where *Y_pre_* and *Y_exp_* are the predicted and experimental response, respectively, and *n* is the number of experiments.

## 4. Conclusions

The present study demonstrates for the first time that immobilized *C. antarctica* lipase (Novozym^®^435) is able to synthesize 2-ethylhexyl ferulate (2-EF). A Box-Behnken design with RSM and ANN was selected to model and optimize the biocatalysis. Both RSM and ANN models for the 2-EF synthesis were built, and ANN was better than RSM in data fitting and prediction. The optimum synthesis condition modeled by ANN was in a reaction time of 24 h, reaction temperature of 78 °C and enzyme amount of 1485 PLU. The conversion of 2-EF under this optimum condition was verified to be 99.74%. The reduced pressure evaporation system combined with solvent free reaction conditions can decrease the reaction time and reaction temperature for ferulic acid ester synthesis.

## Figures and Tables

**Table 1:** Box-Behnken design and observed experimental data for 3-level-3-factor response surface analysis.

Treatment No. ^a^	Experimental Variables			Observed Molar Conversion (%)
	X_1_ (h)	X_2_ (PLU)	X_3_ (°C)	
1	16	500	80	85.7 ± 3.4
2	16	1000	70	92.4 ± 3.1
3	16	1000	70	92.0 ± 2.8
4	16	1000	70	91.7 ± 2.3
5	8	500	70	68.7 ± 4.1
6	24	500	70	82.3 ± 3.2
7	8	1500	70	80.4 ± 2.1
8	24	1500	70	98.8 ± 1.6
9	16	500	60	39.8 ± 2.7
10	8	1000	60	42.7 ± 3.3
11	16	1500	60	44.8 ± 4.2
12	16	1500	80	98.9 ± 2.5
13	24	1000	60	64.2 ± 2.1
14	8	1000	80	86.3 ± 2.8
15	24	1000	80	98.7 ± 1.9

^a^ The treatments were run in a random order.

**Table 2:** ANOVA for synthetic variables pertaining to response percent molar conversion.

Source	Sum of Squares	Degree of Freedom	Prob > F ^a^
*Model*	22.50	9	0.0005
*Linear*			
X_1_	1.87	1	0.0033
X_2_	0.85	1	0.0614
X_3_	14.72	1	<0.0001
*Interaction*			
X_1_X_2_	0.01	1	0.7297
X_1_X_3_	0.17	1	0.1762
X_2_X_3_	0.02	1	0.5973
*Square*			
X_1_^2^	0.02	1	0.6545
X_2_^2^	0.79	1	0.0188
X_3_^2^	4.33	1	0.0005
Lack of fit	15.20	3	0.09
Pure error	0.99	2	
*Total error*	16.19	5	

^a^ Prob > F: level of significance.

**Table 3:** Validation and comparison of RSM- and ANN-modeling biocatalysis.

Run	Independent Variable			Molar Conversion (%)		
	X_1_ (h)	X_2_ (PLU)	X_3_ (°C)	Experimental	RSM Predicted	ANN Predicted
1	14	1250	75	93.2	98.8	95.0
2	15	750	65	76.8	71.2	73.5
3	10	850	75	74.6	90	85.3
4	9	1400	65	65.4	65.1	62.2
5	20	950	60	51.7	56.9	56.3
				AAD	10.046	9.430
				RMSE	7.322	5.152

**Table 4:** The optimal trial obtained from RSM and ANN models.

Model	Optimal Condition			Relative Conversion (%)	
	X_1_ (h)	X_2_ (PLU)	X_3_ (°C)	Experimental Value	Predicted Value
RSM	23	1422	71	99.43	102.74
ANN	24	1485	78	99.74	99.82

**Table 5:** Coding of experimental parameters and related levels.

Parameters	Symbol	Coded Levels		
		−1	0	1
Reaction time (h)	X_1_	8	16	24
Enzyme amount (PLU) ^a^	X_2_	500	1000	1500
Reaction temperature (°C)	X_3_	60	70	80

^a^ PLU means propyl laurate units.
